# Quality of Life, Wishes, and Needs in Women with Gestational Diabetes: Italian DAWN Pregnancy Study

**DOI:** 10.1155/2012/784726

**Published:** 2012-04-26

**Authors:** A. Lapolla, G. Di Cianni, A. Di Benedetto, I. Franzetti, A. Napoli, L. Sciacca, E. Torlone, L. Tonutti, E. Vitacolonna, D. Mannino

**Affiliations:** ^1^Department of Medicine, University of Padova, Italy; ^2^Department of Diabetes and Metabolic Diseases Livorno, ASl6, Livorno, Italy; ^3^Department of Internal Medicine, Messina University, Italy; ^4^UOS Diabetology, Varese Hospital, Italy; ^5^Department of Clinical and Molecular Medicine, S. Andrea Hospital, Sapienza University of Rome, Italy; ^6^Department of Clinical and Molecular Biomedicine Endocrinology Section, University of Catania, Italy; ^7^Department of Internal Medicine, Endocrinology and Metabolism, Santa Maria della Misericordia Hospital, Perugia, Italy; ^8^Department of Endocrinology and Metabolism, Udine University, Italy; ^9^Department of Medicine and Ageing, University of Chieti, Italy; ^10^Department of Endocrinology and Diabetology, Hospital Bianchi Malacrino Morelli, Italy

## Abstract

The DAWN (Diabetes Attitudes, Wishes and Needs) study is a survey promoted by the International Diabetes Federation to recognize the perceptions and attitudes of people suffering from diabetes mellitus. In this context, we evaluated the quality of life of Italian and immigrant women with gestational diabetes mellitus (GDM). Information was gathered using a structured questionnaire for patients' self-compilation. In a 3-month period, a 51-item questionnaire was submitted to 198 Italians and 88 immigrants (from 27 different foreign nationalities). Italian women were older and had higher education than the immigrants. 60% of the Italians and 38% of the immigrants had a family history of diabetes mellitus. In both groups, the diagnosis of GDM caused anxiety; one-third of women feared their child could contract diabetes at delivery and/or have congenital malformations. Some women had trouble in following treatment regimens: the major concern being dietary advice and blood glucose testing. Most women were satisfied (34%) or highly satisfied (60%) with the quality of care, although the degree of cooperation between diabetes specialists and gynaecologists was considered sometimes unsatisfactory. In order to optimize maternal and foetal outcomes, educational projects and improved communication between patients and the healthcare provider team are recommended.

## 1. Introduction

The DAWN study (Diabetes Attitudes, Wishes and Needs) is a survey promoted by the International Diabetes Federation, with the aim of recognizing the feelings and attitudes of patients suffering from diabetes mellitus and the healthcare professionals responsible for patient management and care. Up to now the results of the DAWN study have shown that about half of the people with diabetes have a lower quality of life. The most important factors predicting discomfort or worse quality of life among diabetic patients are the country of residence and, subsequently, the type of healthcare system adopted there (i.e., the presence or absence of a specialized team dedicated to the care and assistance for diabetic patients and their chronic complications) [1, 2].

Inside the Italian DAWN study framework, the Italian DAWN Study Group on Pregnancy performed this survey to evaluate the wishes and needs of Italian and immigrant women affected by GDM. 

## 2. Materials and Methods

### 2.1. Study Design

The research was conducted in 10 Italian centres specialized in the care of pregnant women with diabetes. Each centre supplied 20 questionnaires submitted to pregnant women with GDM by nurses who had previously been trained in this task during a specific briefing session. Overall, 198 questionnaires were collected and analyzed.

The project was also targeted at immigrant women with GDM. The questionnaire used was based on the same criteria as those ones used for Italian women, with the addition of seven questions about the specific condition experienced by immigrants. A Cultural Mediator helped immigrant pregnant women with language difficulties. Overall, 88 questionnaires were collected. The two surveys were conducted in the same period.

The 51 questions covered the following topics:

- the general characteristics of women with GDM,
- the feelings related to the diagnosis of diabetes during pregnancy,
- the evolution of diabetes,
- the diet in pregnancy,
- the family support,
- the gestational diabetes specialist centre,
- the relationship with medical doctors.

The study was approved by the local ethics committee and the women gave their informed consent to participation in accordance with the Declaration of Helsinki.

GDM was diagnosed according to the Carpenter and Coustan's criteria [3]. Women with GDM were helped to achieve good metabolic control: fasting plasma glucose (FPG) <5.3 mM and 2 h postprandial plasma glucose (PPG) <6.7 mM. Patients were given dietary advice about the nutritional requirements of pregnancy [1], and nurse educators showed them how to self-monitor blood glucose. They attended the centres every two weeks for specialist consultations. Insulin treatment was started when FPG was higher than 5.3 mM and/or 2 h PPG higher than 6.7 mM [4]. All women with GDM were monitored for metabolic and obstetric purposes until delivery.

### 2.2. Statistical Evaluation

Data are expressed as absolute values, percentages, or mean ± Standard Deviation (SD). Differences among groups were tested using the chi square or F-test. Statistical significance was set at *P* < 0.05. Significant differences obtained using the F-test were confirmed by the Mann-Whitney *U*-test if the assumptions of the F-tests were not met. All statistical analyses were performed using SPSS statistical package, version 16.0 (SPSS, Chicago, IL).

## 3. Results

### 3.1. General Characteristics

General characteristics of Italian and immigrant women with GDM are reported in Table 1.

The immigrant respondents belonged to as many as 27 different foreign nationalities; the countries of origin with the highest representation were Romania and Morocco, followed by Bangladesh, Albania, and Nigeria. 39% of the immigrant women with GDM lived in Italy for 5–9 years, 30% for 3-4 years, 22% for 1-2 years, and 12.5% for 10–14 years.

### 3.2. Pregnancy and Diabetes

A previous pregnancy complicated by GDM was reported in 25% of Italian women and 42% of Immigrant ones (*P* < 0.005). In 88.5% of immigrant cases, the previous GDM was diagnosed in Italy.

The time of diagnosis of GDM in current pregnancy was made mostly (58.5%) between the 25th–29th week of gestation, although an early diagnosis (before 20 weeks of gestation) was performed in 40.5% of the immigrant women and 27% of the Italian women (*P* < 0.05). Like the Italian respondents, about 90% of the immigrant women were advised to undergo tests to diagnose gestational diabetes by their gynaecologist.

#### 3.2.1. Feelings Arising from the Diagnosis of Diabetes during Pregnancy

The diagnosis of diabetes resulted in the development of anxiety in the same way as in both immigrant women and Italian women (90% versus 87%, *P* = ns). The most predominant feeling among pregnant women was the fear of possible consequences for their child (66%), while a significant minority (28.9%) was also worried about possible malformations in their babies.

**Figure 1:**
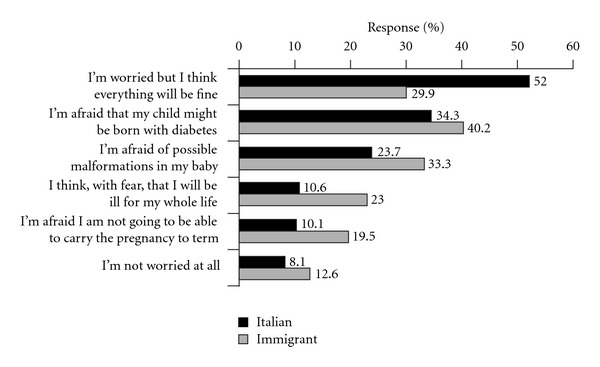
Patients' current concerns in relation to pregnancy. (The sum exceeds 100% because multiple answers were possible). The differences between Italian and Immigrant pregnant women are significant (*P* < 0.05).

Although worried about their general health, 52% of Italian respondents were moderately optimistic throughout their pregnancy. In fact, the frequency of respondents concerned about the consequences for their child (including malformation) decreased, being the mother's main care that of a serious illness or a possible failure in her pregnancy. In contrast, a higher number of immigrant respondents were anxious about their general conditions and about the consequences for their child, and fewer of them were moderately optimistic, (*P* < 0.05), (Figure 1).

#### 3.2.2. Difficulties Related to Diabetes Monitoring

A considerable number of respondents claimed that they had experienced some difficulties in following the dietary advice, home blood glucose, and physical activity recommendations. The initiation of insulin therapy, that occurred in 31.5% of Italian and 40% of immigrant women, increased women's anxiety because they believed that insulin might cause problems for their unborn child (Table 2).

In all respondents, the treatment costs were not a load for the family budget.

**Figure 2:**
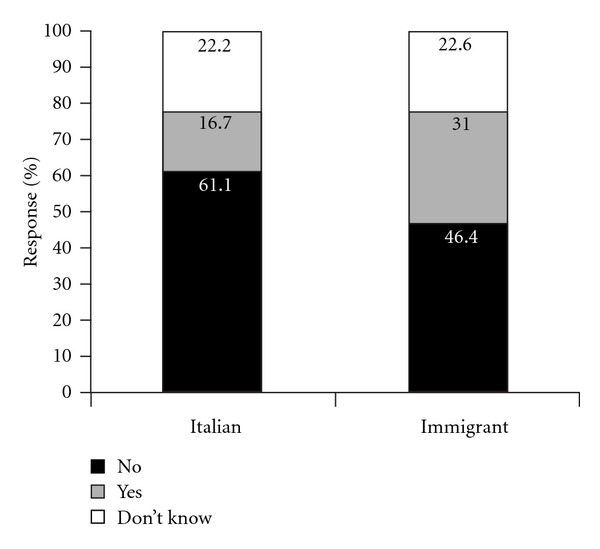
Patients' opinions about whether diet was different from that of pregnant women without diabetes. The differences between Italian and immigrant pregnant women are significant (*P* < 0.02).

The great majority of pregnant women with diabetes did not think that their diet was different from that of pregnant women without diabetes; however, a significantly higher proportion of immigrant women with GDM thought that the diets were indeed different (31% versus 16.7%; *P* < 0.02), (Figure 2). The reasons for this belief can be ascribed to their own experience and comparison with pregnant friends without diabetes, as well as to opinions held within their family, traditional beliefs, and a certain degree of perceived “suffering” from having to follow strict eating patterns and to abstain completely from sugar and sweets. In this respect, it is noteworthy that 78% of the immigrant respondents changed their eating habits nonetheless, and even though 80% of them said they had easy access to shops selling ethnic foods, the majority (58%) had a mixed diet that combined the cuisine of their country with Italian eating habits. 25% of the immigrant women, mostly Muslim, said that there are religious recommendations about diet during pregnancy, but only one third of these women actually put these ones into practice.

95% of Italian women and only 29% of immigrants (*P* < 0.001) felt that their husband and other family members (parents, parents-in-law, sisters, etc.) helped them to deal with the problems related to their pregnancy. Among immigrant women, 40% said that they were helped by other family members, 30% by friends of the same country, 13% by Italian friends, 10% by Cultural Mediator, and 7% by a social worker.

### 3.3. The Gestational Diabetes Specialized Centre and the Relationship with Medical Doctors

**Figure 3:**
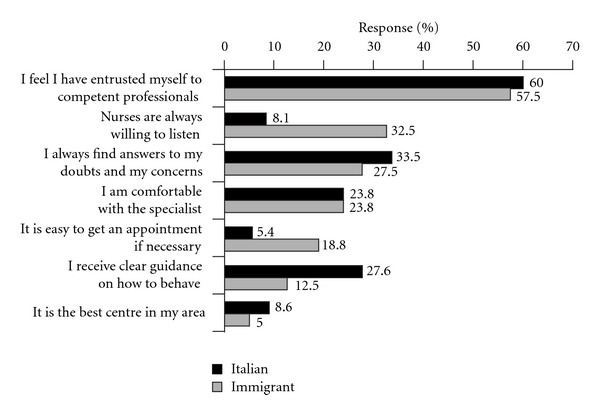
Relationship between patients and diabetes centre. (The sum exceeds 100% because multiple answers were possible).

The majority of respondents (83%) said they had heard about the Diabetes Centre from their gynaecologist; only 3.5% of Italian women and 23.5% of immigrant women received this information from their general practitioner. Most respondents were satisfied (34%) or highly satisfied (60%) for the quality of care provided by Diabetes Centres (Figure 3).

Pregnant women were usually comfortable talking to their gynaecologist and diabetes specialists. Among immigrant respondents, 8% declared some difficulties to have an appointment for gynaecologist consultancy and 13% did not have a gynaecologist they could trust. Gynaecologists and diabetes specialists cooperated in only 25-26% of cases, and most pregnant women (73%) felt that better cooperation between these practitioners is the best way to improve the care available to pregnant women with diabetes.

## 4. Discussion

The data from this survey, which is the first conducted in the field of gestational diabetes, has highlighted several areas of concern.

The diagnosis of a chronic disease, for example, diabetes mellitus, may generate anxiety [5]; this is especially true for women who received the diagnosis of diabetes during their pregnancy [6, 7]. An effective and satisfactory communication between pregnant women with gestational diabetes and healthcare providers can be fundamental to reduce their level of anxiety. These women need information about the disease, the potential risks for mother and child, the management strategy, and a treatment plan to avoid maternal and foetal complications [8].

In our study population, all women received structured information from a healthcare team comprising diabetologists, experienced nurses and dieticians. This results in relatively few fears related to GDM, and in a moderate degree of optimism in many women. Thus, the role of the care team is crucial for immigrant women with GDM, particularly when considering their possible language difficulties, as well as cultural and religious differences [9–11].

Most women were satisfied with the treatment received at the Diabetic Centre for the competence of the healthcare team and the availability of the experienced nurses to listen to their doubts and worries and to help them to overcome their concerns. Nevertheless, it should be emphasized that a great number of women regarded the degree of cooperation between diabetes specialists and gynaecologists as unsatisfactory. We found that gynaecologists and diabetes specialists cooperated in about 25% of cases. Therefore, it was not surprising to find that most pregnant women indicated greater contact between these practitioners as the main way through which to improve the care of women with GDM. If you consider the difficulties that immigrant women encountered in contacting or being visited by their gynaecologist, it is essential that general practitioners should play a more crucial role in the care of pregnancy complicated by diabetes throughout the gestation period, starting with the diagnostic procedures and, finally, in the preconception period.

This survey shows that even in centres that have reached a good standard for treatment of pregnant women, GDM is often diagnosed at a late stage of pregnancy, confirming recently published Italian data [12]. Finally, the presence of an increasing number of women from foreign countries contacting diabetes centres during their pregnancies suggests a need for dedicated practices, with specially trained personnel, to ensure that treatment regimens are well adopted [10].

The limitations of this study are that the maternal and foetal outcomes of pregnant women were not considered and the results were obtained only from selected centres specializing in the care of diabetes during pregnancy in Italy (most small diabetes centres are not included in this survey). Nevertheless, the strength of the survey is that, to the best of the authors' knowledge, no other similar study has been published so far. The data have allowed us to identify some care aspects that need to be examined in order to improve the quality of life of women with GDM and reduce the related foetal-maternal morbidity. To achieve this outcome, it is essential to carry out information campaigns directed to young women, using the press and other mass media, to raise awareness of the issues such as screening, diagnosis, and postpartum followup of diabetes mellitus.

Furthermore, immigrants make up a significant proportion of the pregnant population in Italy, as well as in other European countries [13], so it is necessary to promote training initiatives for the establishment of regional networks to improve communication between patients, cultural mediators, and healthcare professionals. In conclusion, measures to improve communication and cooperation between the various healthcare professionals will be essential if local and immigrant women with GDM are to deliver healthy, full-term babies with minimal risk to themselves.

## Figures and Tables

**Table 1:** General characteristics of women with GDM.

	Italians	Immigrants
Cases (*n*)	198	88
Age (years)	34.2 ± 5	32.5 ± 4*
Primiparous (%)	50.5	44*
Married (%)	92	95
Single/Divorced (%)	8	5
*School level *		
University/High School (%)	72	24*
Primary/Middle (%)	28	76*
*Occupation *		
Office workers (%)	34	—
Housewife (%)	20	52
Factory workers (%)	18	10
Shop assistant (%)	14	—
Housemaid (%)	2	16
Caregiver for elderly (%)	—	5
Unemployed (%)	6	12
Other occupations (%)	6	5

*P < 0.05.

**Table 2:** Difficulties and anxiety related to diabetes monitoring.

	Italians	Immigrants
*Difficulty in following:*		
Dietary advice (%)	58	43.5*
Home blood glucose (%)	63	36.5°
Physical activity (%)	50.5	61
Increase in anxiety for insulin therapy	31.5	40

*P < 0.02; °*P* > 0.01.

## Abbreviations

GDM: Gestational diabetes mellitus
DAWN: Diabetes Attitudes, Wishes and Needs
GCT: Glucose challenge test
OGTT: Oral glucose tolerance test
FPG: Fasting plasma glucose
PPG: Postprandial plasma glucose
SD: Standard deviation.
